# Book Reviews

**Published:** 1892-11

**Authors:** 


### *BOOK REVIEWS.*

An American Text-Book of Surgery. Edited by William W. Keen, M. D., and J. William White, M. D., with the assistance of Drs. C. H. Burnett, P. S. Connor, F. S. Dennis, Roswell Park, L. A. Stimson, L. S. Pilcher and others. W. B. Saunders, 913 Walnut street, Philadelphia.

This is the most recent contribution to surgical literature, and, as its name implies, is a system by American authors who are recognized as leaders in the surgical field.

Though it is impossible to notice in detail the features of the work, we feel safe in saying that any one who glances over the volume must be impressed with the value of all of the contributions, and in many subjects the matter is brought down to a more recent date than can be found in any other work. Among others we especially note the chapter on surgery of the brain, the opening article on surgical bacteriology and that on intestinal surgery.

The illustrations are of a high character, representing colored plates of bacilli, many reproductions of photographs, and numerous wood-cuts. Altogether this part of the work will take a high rank.

One feature that is to be much regretted is that there is no means of knowing the writer of each article, the publisher having contented himself with an "omnibus" list of the authors at the beginning of the work. It is undoubtedly true that most men like to know after whom they are reading, and in this respect the work will be a disappointment. But, as a whole, it is deserving of the highest commendation.

---

Encyclopædic Medical Dictionary—A dictionary of technical terms used by writers on medicine and the collateral sciences. By Frank P. Foster, M.D., Editor of the *New York Medical Journal*, and Librarian of the New York Hospital. Vol. 3. Sold only by Subscription. D. Appleton & Co., New York.

This large work has been in preparation by Dr. Foster and his able staff of collaborators for several years. The two volumes which have previously appeared have made the profession so well acquainted with its merits that an elaborate review will not be necessary. Many dictionaries have appeared of late, varying in worth as well as in size, but to Dr. Foster and his faithful assistants, will belong the honor of furnishing the one which, for many years at least, will be our final appeal and highest authority. The present is the third volume. The character of the book imposes a difficult task upon the reviewer. The work has its excellencies and its faults. Among the latter is the space devoted to the botanical description of plants at the expense of physiological and therapeutic relations. Columns are occupied with directions for making the several preparations of drugs, tinctures, infusions, extracts, etc., but not one word said about their uses or even their doses. It seems a little inappropriate also that *Laurel* should occupy a column, and *Hysteria* only half that space; that *Hammah* (an Arabian prefix to certain bathing places) should be given as much space as *Hemorrhage;* that the description of *Gleet* should be dismissed in two lines while that of *Glanders* occupies nearly a column.

The volume is supplied with numerous excellent illustrations, and the mechanical work has been done faultlessly. The completed work (there will be one more volume) will be a monument to the painstaking labor of the editors, and such a valuable contribution to medical and scientific terminology will receive wide-spread encouragement and support.

